# *Elaeagnus angustifolia* Plant Extract Inhibits Epithelial-Mesenchymal Transition and Induces Apoptosis via HER2 Inactivation and JNK Pathway in HER2-Positive Breast Cancer Cells

**DOI:** 10.3390/molecules25184240

**Published:** 2020-09-16

**Authors:** Ayesha Jabeen, Anju Sharma, Ishita Gupta, Hadeel Kheraldine, Semir Vranic, Ala-Eddin Al Moustafa, Halema F. Al Farsi

**Affiliations:** 1 College of Medicine, QU Health, Qatar University, Doha P.O. Box 2713, Qatar; jabeen@qu.edu.qa (A.J.); anju.sharma7385@gmail.com (A.S.); ishugupta28@gmail.com (I.G.); hk1805332@student.qu.edu.qa (H.K.); svranic@qu.edu.qa (S.V.); 2 Biomedical Research Centre, Qatar University, Doha P.O. Box 2713, Qatar; 3 College of Pharmacy, Qatar University, Doha P.O. Box 2713, Qatar

**Keywords:** *Elaeagnus angustifolia*, breast cancer, EMT, chemoprevention, apoptosis

## Abstract

*Elaeagnus angustifolia* (*EA*) is a medicinal plant used for treating several human diseases in the Middle East. Meanwhile, the outcome of *EA* extract on HER2-positive breast cancer remains nascent. Thus, we herein investigated the effects of the aqueous *EA* extract obtained from the flowers of *EA* on two HER2-positive breast cancer cell lines, SKBR3 and ZR75-1. Our data revealed that *EA* extract inhibits cell proliferation and deregulates cell-cycle progression of these two cancer cell lines. *EA* extract also prevents the progression of epithelial-mesenchymal transition (EMT), an important event for cancer invasion and metastasis; this is accompanied by upregulations of E-cadherin and β-catenin, in addition to downregulations of vimentin and fascin, which are major markers of EMT. Thus, *EA* extract causes a drastic decrease in cell invasion ability of SKBR3 and ZR75-1 cancer cells. Additionally, we found that *EA* extract inhibits colony formation of both cell lines in comparison with their matched control. The molecular pathway analysis of HER2 and JNK1/2/3 of *EA* extract exposed cells revealed that it can block HER2 and JNK1/2/3 activities, which could be the major molecular pathway behind these events. Our findings implicate that *EA* extract may possess chemo-preventive effects against HER2-positive breast cancer via HER2 inactivation and specifically JNK1/2/3 signaling pathways.

## 1. Introduction

Breast cancer (BC) commonly affects women worldwide, comprising 25% of cancer cases [1]. There are several risk factors, both environmental and genetic, associated with the onset of breast cancer [2]. Gene-expression-profiling studies classified breast cancer into five molecular subtypes: Luminal (A and B), HER2, basal-like, and normal-like, using hierarchical cluster analysis [3]. Among all subtypes, HER2-positive breast cancer accounts for 20–25% and is associated with aggressive phenotype, poor prognosis, and survival rate, in addition to increased recurrence [4]. Systemic management modalities for HER2-positive breast cancer include chemotherapy, radiation, and targeted anti-HER2 treatment modalities [4,5,6,7]. Although the treatment is generally effective in the early stages of therapy, nevertheless, ~90% of primary and half of metastatic breast cancer cases resistance to therapy leads to treatment failure and mortality [8]. Thus, it is important to identify novel potent therapeutic agents that can inhibit cell proliferation of HER2+ breast cancer with minimal side-effects. An alternate to conventional therapy can be found in naturally present phytochemicals in foods such as vegetables, fruits, spices, and plant roots [9,10]. Traditionally, *Elaeagnus angustifolia* (*EA*) plant has been used extensively for centuries in the treatment of various diseases due to its antioxidant, anti-inflammatory, and antimicrobial properties [11], along with bioactive compounds (flavonoids and coumarins) [12] that regulate key events associated with cancer development, such as cell-signaling pathways, including Wnt-signaling, cell proliferation, cell-cycle progression, apoptosis, and epithelial-to-mesenchymal transition (EMT) [13,14].

*Elaeagnus angustifolia* (*EA*), commonly known as wild olive, oleaster, silver berry, or Russian olive [11,15], is a deciduous tree belonging to the family of *Elaeagnacea* (Araliaceae) widely distributed in the Middle East, as well as Mediterranean regions [12,16]. *EA* fruit is highly nutritious and contains vitamins (vitamin C, tocopherol, thiamine B1, and carotene), sugar, proteins, and several minerals, like potassium, iron, magnesium, and calcium [17,18,19]; the leaves and flower extract are rich in secondary metabolites such as coumarins, phenolcarboxylic acids, flavonoids, saponins, and tannins [16,20,21]. Previous studies have shown that *EA* exhibits anticancer effects as a result of its essential oils (ethyl cinnamate, 2-phenyl-ethyl benzoate, 2-phenyl-ethyl isovalerate, nerolidole, squalene, and acetaphenone), flavonoids (quercetin), and pro anthocyanosides [22,23]. In cancer, flavonoids are shown to enhance p53 expression and cause cell-cycle arrest in the G2/M phase [24]. Moreover, they are known to inhibit Ras protein expression and regulate heat-shock proteins in various cancers, mainly in leukemia and colorectal cancer [24]. One of the key flavonoid components of *EA* is Quercetin, which is an anti-proliferative agent [24]. Furthermore, quercetin also promotes TRAIL-induced apoptosis by enhancing the expression of Bax and inhibiting Bcl-2 protein [25,26,27]. Additionally, ethyl acetate has been shown to significantly reduce proliferation of Hela cells in vitro [22]. Apart from this, volatile oils present in the plant have medicinal properties and are also used in perfume industries [23,28]. *EA* possesses numerous therapeutic and pharmacological properties, including antifungal, antibacterial, antimutagenic, anti-inflammation, antioxidant, and gastroprotective effects [11,29,30,31,32]. Traditionally, *EA* is also used to cure other diseases, including osteoporosis, amoebic dysentery, jaundice, asthma, flu, cough, cold, nausea, diarrhea, sore throat, fever, tetanus, and female aphrodisiac [12,15,33,34]. However, there are limited studies regarding the role of *EA* extract on cancer. In this context, our group recently demonstrated that *EA* extract can reduce the progression of human oral cancer by the inhibition of angiogenesis and cell invasion via Erk1/Erk2 signaling pathways [12].

A previous study showed that hydroalcoholic extracts of *EA* flower significantly inhibit angiogenesis, one of the known hallmarks of cancer [35]. Nevertheless, there are no studies reported on the anticancer activity of *EA* in breast cancer, especially in HER2-positive type, and its mechanism of cancer inhibition. To investigate the potent therapeutic and antitumor properties of *EA* extract in human breast cancer and its underlying mechanism, we explored the effect of aqueous extract of *EA* flower on cell proliferation and cell-cycle progression, cell invasion, and colony formation in two HER2-positive human breast cancer cell lines (SKBR3 and ZR75-1).

## 2. Results

**Figure 1:**
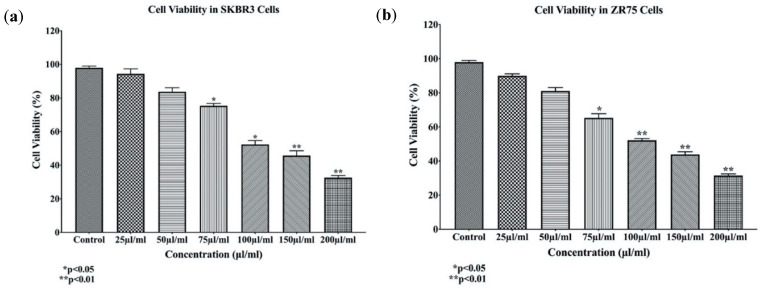
(**a**,**b**) The effects of different concentrations of *Elaeagnus angustifolia (EA)* plant extract on cell proliferation of HER2-positive breast cancer cell lines SKBR3 (**a**) and ZR75-1 (**b**) at 48 h. Data indicate an inverse relation between concentrations of *EA* extract and cell proliferation in both SKBR3 and ZR75-1 cell lines. Data are expressed as percent of growth ± SEM.

In order to determine the effects of *EA* extract on HER2-positive cell lines SKBR3 and ZR75-1, cells were treated with varying concentrations of *EA* extract (25, 50, 75, 100, 150, and 200 µL/mL) for 48 h. Treatment with EA extract reduced the number of proliferating HER2-positive breast cancer cells in a dose-dependent manner (Figure 1); notably, concentrations of 100 and 200 µL/mL showed a substantial decrease in cell viability of SKBR3 and ZR75-1 by 50% and 75%, respectively.

**Figure 2:**
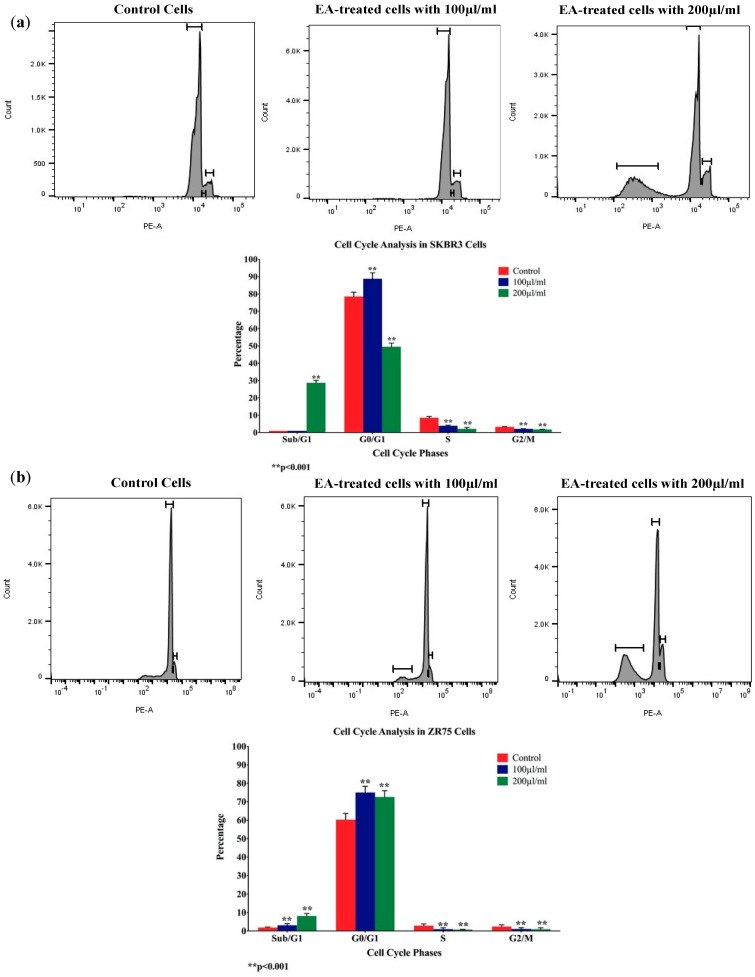
(**a**,**b**) Flow cytometry data analysis of SKBR3 and ZR75 cells after *EA*-treatment. Data demonstrate an increase in G_0_/G_1_ phase with simultaneous reduction in S and G_2_/M phases in both cell lines. Meanwhile, there is a significant increase in cell apoptosis (Sub/G_1_ phase) of SKBR3 cells treated with *EA*, and a small increase in cell apoptosis of treated ZR75 cells.

Meanwhile, and to examine whether the antiproliferative effect of the *EA* flower extract on SKBR3 and ZR75-1 cells is associated with cell-cycle deregulation, we analyzed cell-cycle phase distributions of *EA*-treated cells, using flow cytometric analysis. Our results showed that exposure to *EA* extract (100 and 200 µL/mL) for 48 h enhanced the G_0_/G_1_ phase, with a simultaneous decrease in S and G_2_/M phases of both the breast cancer cell lines, thus indicating *EA*-induced cell-cycle inhibition (Figure 2). Furthermore, we observed a significant increase in the sub/G_1_ phase of both cell lines, indicating that cells undergo apoptosis when treated with *EA* extract (Figure 2).

**Figure 3:**
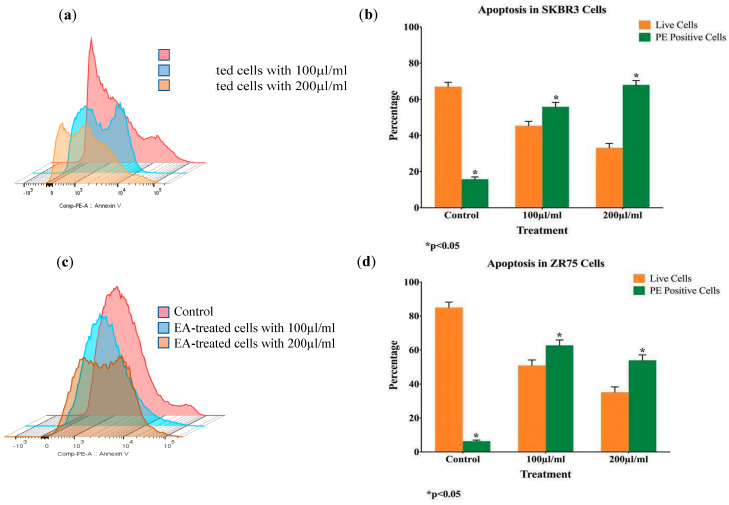
(**a**,**b**) Induction of apoptosis by *EA* extract in SKBR3 (**a**, **b**) and ZR75 (**c**, **d**) cells, as determined by Annexin V-FITC and 7-AAD apoptosis assay.

To confirm *EA*-induced apoptosis, Annexin V-FITC and 7-AAD staining by flow cytometry were performed. Therefore, the presence of apoptosis is clearly demonstrated in both cell lines (Figure 3).

**Figure 4:**
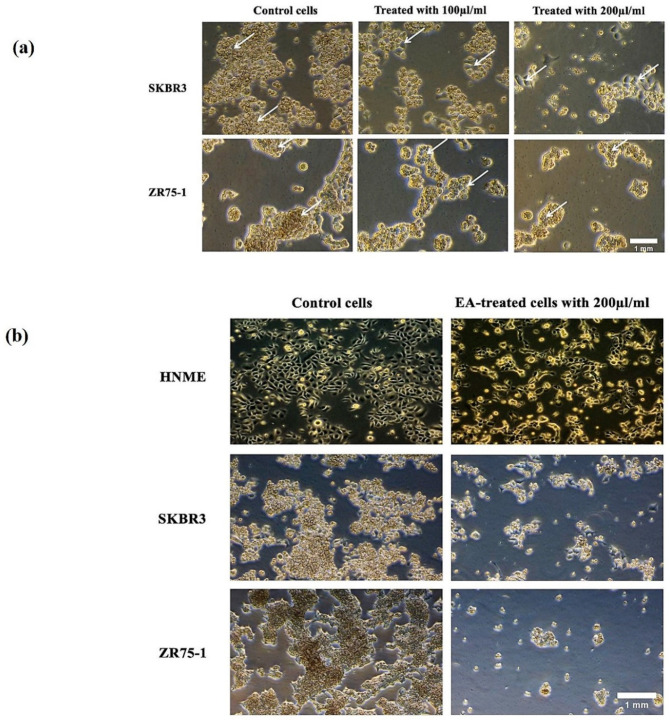
(**a**,**b**) *EA* plant extract induces morphological changes in HER2-positive cell lines, SKBR3 and ZR75-1. (**a**) We observe that treatment for 48 h with 100 and 200 μL/mL of *EA* extract induces epithelial transition and the formation of a monolayer of cells in both cell lines, in comparison with untreated (control) cells which display a round phenotype and form multilayers; arrows indicate epithelial morphology with clear cell-cell adhesion. (**b**) At three days of treatment of SKBR3, ZR75-1, and HNME-E6/E7 cell lines with 200 μL/mL of *EA* plant extract, the two cancer cell lines start detaching from the surface of the tissue culture dish, indicating cell death; this observation was not noted in the HNME-E6/E7 cells (images **a** and **b** at ×20 magnification).

Next, we examined the cell morphology of SKBR3 and ZR75-1 in addition to HNME-E6/E7 cell lines, using phase-contrast microscopy, under the effect of 100 and 200 μL/mL of *EA* extract. In the absence of treatment, SKBR3 and ZR75-1 cells displayed a round morphology and disorganized multilayered cells. In contrast, and as indicated in Figure 4a, treatment for 48 h with 100 and 200 μL/mL of *EA* plant extract led to a phenotypic conversion from round cells to epithelial-like phenotype. Clearly, cells became more flattened in appearance and showed an increase in cell-cell adhesion, in comparison with untreated cells (Figure 4a). However, at three days of treatment with 200 μL/mL of *EA* plant extract, cells started detaching from the surface of the tissue culture dish, indicating cell death in SKBR3 and ZR75-1 cells; however, this was not observed in the human normal immortalized mammary epithelial cell line (HNME-E6/E7), as shown in Figure 4b. Nevertheless, it is evident that *EA* extract inhibits cell proliferation HNME-E6/E7 cells, with a slight effect on their epithelial morphology (Figure 4b).

These results imply that moderate concentrations of 100 μL/mL of *EA* plant extract induce cell differentiation after 24 and/or 48 h, while higher concentrations (200 μL/mL of *EA* plant extract) can provoke apoptosis after 48 h of exposure.

**Figure 5:**
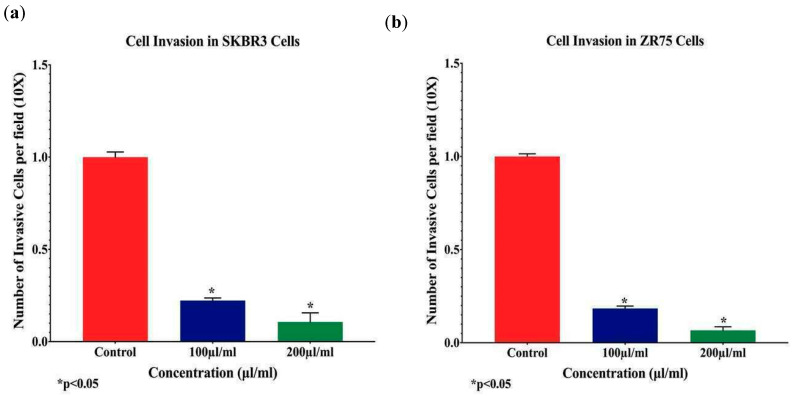
(**a**,**b**): The effects of *EA* flower extract on cell invasion of human HER2-positive breast cancer cells. *EA* extract inhibits cell invasion ability of SKBR3 (**a**) and ZR75-1 (**b**) cell lines by approximately 70% in comparison with their matched control cells (unexposed) (*p* < 0.05). Boyden chambers were used to assess cell-invasion ability of SKBR3 and ZR75-1 cell lines. Cancer cells treated for 24 h with 100 and 200 µL/mL *EA* plant extract showed a significant inhibition of cell invasion in both cell lines, when compared with their matched control (*p* < 0.05). Data are quantified by normalizing the number of invasive cells by their total number.

Subsequently, and to analyze the anti-invasion effects of *EA* on HER2-positive breast cancer cells, Matrigel invasion assay was performed, using SKBR3 and ZR75-1 cells, upon *EA* treatment with 100 and 200 μL/mL concentrations; our data revealed that *EA* extract significantly inhibits cell invasion ability of both cell lines by ~70% to 88%, respectively, in comparison with control cells (Figure 5, *p* < 0.05). This suggests that *EA* plant extract can considerably downgrade cell invasion and metastasis of HER2-positive breast cancer.

**Figure 6:**
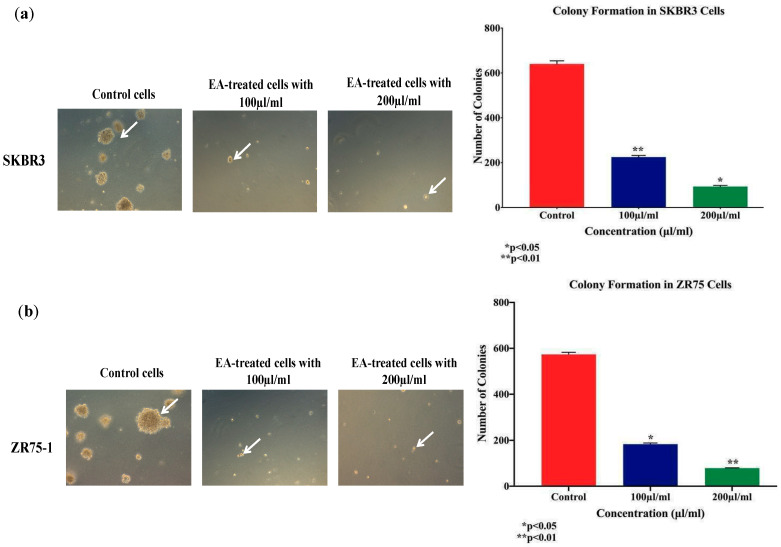
(**a**,**b**) Effect of *EA* flower extract on colony formation, in soft agar, in human HER2-positive cancer cell lines, SKBR3 (**a**) and ZR75-1 (**b**). *EA* extract inhibits colony formation of SKBR3 and ZR75-1, in comparison with their matched control cells (images of figure **a**,**b** at ×10 magnification). Colony formation in soft agar is a solid indicator of tumor formation in vivo. The colonies were counted manually and expressed as percentage of treatment relative to the control (mean ± SEM).

On the other hand, we assessed the colony formation of SKBR3 and ZR75-1 cells, in soft agar, under the effect of *EA* plant extract at 100 and 200 µL/mL, for two weeks; we observed a significant decrease in the number of colonies for both cell lines treated with *EA* plant extract, compared with their matched control, as shown in Figure 6. SKBR3 sustained significant inhibition of colony formation by 60% (*p* < 0.01) and 80% (*p* < 0.05) when exposed to 100 and 200 µL/mL *EA* plant extract, in comparison to the control, respectively (Figure 6a). In parallel, ZR75-1 cell line also displayed a similar pattern after two weeks of treatment; the number of colonies decreased by 70% (*p* < 0.05) and 85% (*p* < 0.01) at 100 and 200 µL/mL concentrations, respectively (Figure 6b). This indicates that *EA* plant extract suppresses colony formation and probably tumor growth in vivo.

**Figure 7:**
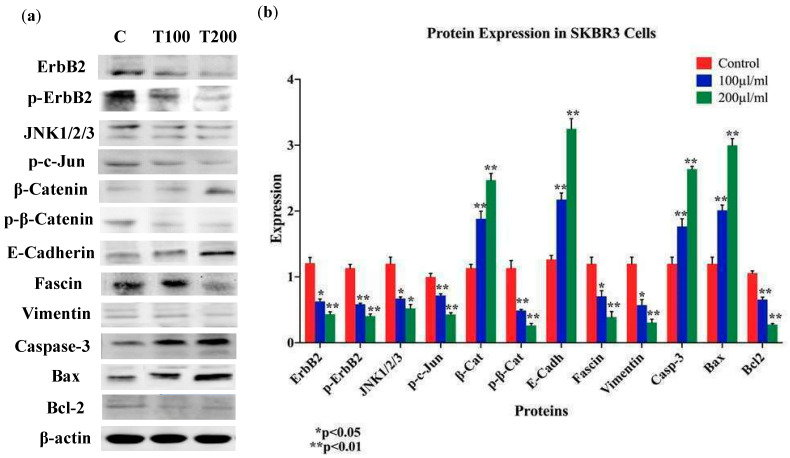
(**a**,**b**) Protein expression and molecular mechanisms of *EA* inhibitory actions in SKBR3 cell line. This plant extract induces an overexpression of E-cadherin, β-catenin, and downregulation of vimentin and fascin, while upregulating pro-apoptotic markers (Bax and Caspase-3), in comparison with their control and inhibiting anti-apoptotic markers (Bcl-2). Furthermore, *EA* plant extract inhibits the phosphorylation of ErbB2 and β-catenin, as well as the expression of JNK1/2/3. β-actin was used as a control for the proteins amount in this assay. Cells were treated with 100 and 200 μL/mL of *EA* extract for 48 h, as explained in the materials and methods and the results sections. (**a**) Blot image and (**b**) quantification of bands.

**Figure 8:**
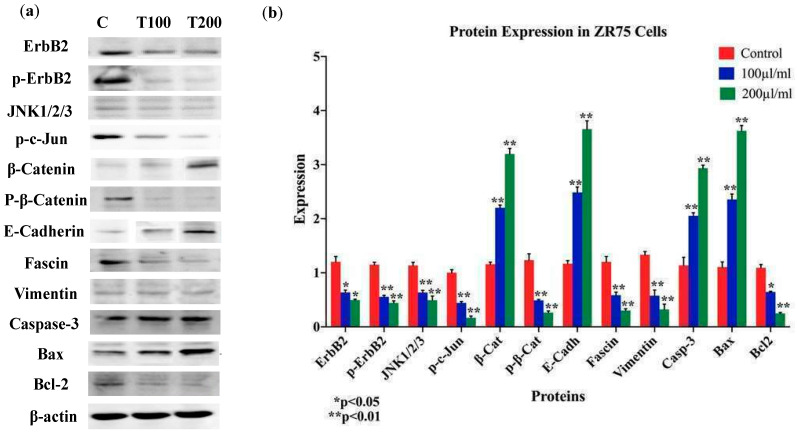
(**a**,**b**) Protein expression and molecular mechanisms of *EA* inhibitory actions in ZR75 cell line. This plant extract induces an overexpression of E-cadherin, β-catenin, and downregulation of vimentin and fascin; in addition, pro-apoptotic markers Bax and Caspase-3 are upregulated in comparison with their control, while anti-apoptotic marker Bcl-2 is inhibited. Furthermore, *EA* plant extract inhibits the phosphorylation of ErbB2 and β-catenin, as well as JNK1/2/3 expression. β-actin served as a control in this assay. Cells were treated with 100 and 200 μL/mL of *EA* extract for 48 h, as explained in the materials and Methods section. (**a**) Blot image and (**b**) quantification of bands.

Based on the above data, we explored the expression patterns of key markers of EMT and cancer progression: E-cadherin, β-catenin, vimentin, and fascin; our data pointed out that *EA* extract enhances the expression of E-cadherin and β-catenin in SKBR3 and ZR75-1 cell lines, while the expression of vimentin and fascin are decreased in comparison to their control cells (Figure 7 and Figure 8). In parallel, we examined the outcome of *EA* on pro-apoptotic proteins (caspase-3 and Bax) and anti-apoptotic protein (Bcl-2) with 100 and 200 μL/mL of *EA* plant extract after 24 and 48 h of exposure. We found enhanced expression of both pro-apoptotic proteins (Bax and caspase-3) in SKBR3 and ZR75-1 in *EA*-treated cells, compared to their control (Figure 7 and Figure 8). In contrast, the expression of Bcl-2 was lost in SKBR3 and ZR75-1 (Figure 7 and Figure 8). Our data suggest that high concentrations of *EA* induce apoptosis in HER2-positive cancer cells, which might be associated with the Bcl-2/Bax/caspase-3 signaling pathway.

Vis-à-vis the underlying molecular pathways of *EA* extract on cell proliferation, EMT progression, cell invasion, and colony formation of HER2-positive breast cancer cells, we assumed that HER2 activation, as well as c-Jun N-terminal kinase (JNK), could have major roles in regulating these events [36,37,38,39]; therefore, the expression patterns of HER2 and JNK1/2 were explored. We found that *EA* extract inhibits the phosphorylation of HER2 (with slight change in its expression level) and β-catenin, while it provokes a downregulation of JNK1/2 in SKBR3 and ZR75-1 upon treatment with *EA* plant extract after 24 and 48 h of exposure (Figure 7 and Figure 8).

## 3. Discussion

In this study, we investigated the effect of *EA* extract in HER2-positive human breast cancer cell lines (SKBR3 and ZR75-1) with regard to certain parameters related to cell proliferation, cell cycle, morphological changes (round to epithelial-like transition: RELT), cell invasion, and colony formation. Additionally, we explored the molecular pathways behind these events. We report that *EA* plant extract can suppress cell proliferation, as well as dysregulate cell-cycle progression of SKBR3 and ZR75-1 cells, along with induction of RELT and inhibition of colony formation in both cell lines. *EA* plant is known for its antioxidant characteristics and has been used conventionally for the treatment of several diseases and inflammation [16,20,21]. Moreover, *EA* consists of bioactive compounds (flavonoids and neoclerodane diterpenoids), which can play a role in promoting apoptosis and cell-cycle progression, as well as inhibiting angiogenesis and EMT events, thus potentially preventing cancer development and progression [12,13,16,40]. Meanwhile, we herein demonstrate that *EA* plant extract inhibits cell proliferation and dysregulate cell-cycle and EMT progression of HER2-positive breast cancer cells.

Indeed, EMT is a crucial phenomenon in cancer progression, characterized by disruption of intracellular tight junctions and the loss of cell-cell contact and epithelial cell features, along with the gain of mesenchymal morphology [41]. On the other hand, it is well-known that cancer progression is characterized by loss of differentiation in human carcinomas, together with downregulation of E-cadherin, which is associated with the degree of tumor malignancy [33]. Moreover, previous studies on different types of human carcinomas have shown that loss of E-cadherin and β-catenin, in addition to enhanced expression of vimentin and fascin, can promote EMT, which is associated with cancer progression [41,42,43,44]. In this investigation, we analyzed the effect of *EA* on E-cadherin, β-catenin, vimentin, and fascin expression patterns in HER2-positive breast cancer cell lines. We observed two different major events that are provoked by *EA* treatment, namely induction of RELT “MET” and apoptosis. More specifically, we found that, upon *EA*-treatment for 24 and 48 h at both low and high concentrations (100 and 200 μL/mL), E-cadherin and β-catenin are upregulated, while vimentin, p-β-catenin, and fascin expressions are downregulated, which are important elements of mesenchymal-epithelial transition (MET), the opposite event of EMT, and RELT. Thus, *EA* plant extract can induce differentiation to an epithelial phenotype and consequently block cell invasion of the two HER2-positive human breast cancer cell lines. On the other hand, we found that *EA* extract inhibits colony formation of SKBR3 and ZR75-1 cell lines, which could be considered as an in vivo tumor formation.

On the other hand, regarding the molecular pathways of *EA* extract on our cell line models, we herein reported that, upon *EA*-treatment after 24 h at both low and high concentration, *EA* extract can inactivate HER2 receptor, as well as deregulate the expression patterns of JNK1/2, which can lead to increased expression of E-cadherin and β-catenin and decreased expression of vimentin and fascin, thus indicating restoration of cell-cell adhesion, especially E-cadherin/catenins complex. Furthermore, in accordance with our data, a study by Wang et al. showed overexpression of JNK to be linked with breast cancer cell migration and invasion, as well as EMT [45]. Therefore, in this present investigation, we show that *EA* plant extract can regulate the RELT/EMT event and inhibit cell invasion of the two human HER2-positive breast cancer cell lines. Moreover, these present data are concurrent with our recently published work regarding the outcome of *EA* plant extract on human oral cancer cells, where we have demonstrated that *EA* induces differentiation to an epithelial phenotype; and therefore, it causes a dramatic decrease in cell invasion and motility of human oral cancer cells, along with an upregulation of E-cadherin expression [12]. Additionally, our study of *EA* extract on oral cancer cells revealed that *EA* can inhibit the phosphorylation of Erk1/Erk2 and β-catenin, which could be behind the initiation of RELT/MET event and the overexpression of E-cadherin [12]. In the present work, we demonstrated that JNK1/2 pathway is one of the main molecular pathways of *EA* in HER2-positive human breast cancer cells.

JNK substrate proteins encompass several nuclear proteins, including transcription factors, as well as nuclear hormone receptors involved in maintaining various cellular activities comprising cell proliferation, differentiation, cell death, and cell survival [46]. JNKs phosphorylate and stimulate both, nuclear and non-nuclear proteins and form the transcription factor activator protein-1 (AP-1) by dimerization of the Jun proteins (c-Jun, JunB, and JunD) with the Fos proteins (c-Fos, FosB, Fra-1, and Fra-2); other downstream molecules include activating transcription factor 2 (ATF-2), c-Myc, p53, STAT1/3, Pax family of proteins, Elk1, NFAT, and Bcl-2 family (Bcl-2, Bcl-xl, Bad, Bim, and Bax) [47]. Of these nuclear substrates, c-jun is the most vital nuclear substrate; JNKs enhance c-jun transcription by binding and phosphorylating c-jun at Ser73 and Ser63 via Ha-Ras, c-Raf, and v-Src [48,49,50]. c-Jun, the downstream target of the JNK pathway, is necessary for Ras-induced carcinogenesis [51,52]. In vivo studies have indicated an oncogenic role of c-Jun in the liver [53,54,55], as well as intestinal cancers [54], thus indicating a pro-oncogenic role for the JNK/c-Jun axis. The activity of c-jun is essential for the Ha-Ras mediating carcinogenesis transformation. Our data indicate that *EA*-treatment reduced p-c-Jun expression, indicating an *EA*-tumor-suppressive role in cancer.

In parallel, it is evident that β-catenin signaling pathways are also involved in these events; this is based on the fact that β-catenin acts as a transcription regulator, as well as cell-cell adhesion molecule, which was elegantly reported by Kandouz et al., under the effect *Teucrium polium* on human prostate cancer cells [12,56]. Thus, it is possible that *EA* plant extract can have a similar effect on β-catenin pathways, especially since our data showed that *EA* extract inhibit β-catenin phosphorylation, consequently allowing it to translocate from the nucleus to undercoat membrane to act as a cell-cell adhesion protein leading to the inhibition of cell-invasion ability of SKBR3 and ZR75-1 cell lines. We surmise that *EA* exhibits anticancer activity due to the high levels of flavonoids, coumarins, and antioxidants [12,14,57].

Vis-à-vis the interaction between the activation of HER2 receptor and its downstream pathways, including JNK, it is well-established that HER2 overexpression causes homo- or heterodimerization, leading to phosphorylation of this receptor, which in turn triggers downstream signaling pathways responsible for important cellular functions, including cell proliferation, invasion, migration, angiogenesis, chemoresistance, and apoptosis [38,39]. We investigated the downstream target of HER2 stimuli, JNK, as JNK-dependent gene regulatory circuitry underlying cell-fate changes from epithelial to mesenchymal state. Our study demonstrates that *EA* slightly suppresses the expression of HER2 receptor, while mostly affecting its phosphorylation, as well as one of its main downstream targets JNK. More specifically, HER2 downregulation is associated with inhibition of proliferation and invasion of HER2-positive human breast cancer cells [58]; this correlates with our results of *EA*-induced decreased cell proliferation, cell invasion, and colony formation.

Regarding the outcome of high-concentration treatment of *EA* (200 μL/mL) after 48 h, we observed induction of apoptosis in *EA*-treated cells by analyzing the mitochondrial apoptosis regulators of Bcl-2 family (Bcl-2 and Bax), as well as Caspase-3 [59]. Bcl-2 homodimers have been shown to inhibit apoptosis; however, Bax homodimers initiates cell death [60]. Heterodimerization between Bax and Bcl-2 and its ratio of Bax to Bcl-2 determine the susceptibility of cells to apoptosis, whereas caspase-3 is known to act as a downstream target of Bax/Bcl-2 control and play a key role in the execution of apoptosis [60]. We herein report that *EA* can reduce the growth and provoke apoptosis of human HER2-positive breast cancer cells. This effect is associated with caspase-3 activation and reduced Bcl-2 expression. Moreover, mitochondrial Bax translocation and the expression of Bcl-2 slightly decreased upon *EA*-extract treatment, indicating that caspase-dependent pathways are involved in *EA*-induced apoptosis and Bcl2/Bax/Caspase-3-regulated cell death through JNK inactivation.

The JNK pathway is predominantly involved in the stimulation of the intrinsic apoptotic pathway facilitated by mitochondria [61]. However, the JNK pathway is also involved in TRAIL-induced apoptosis, autophagy, mitotic catastrophe, and immunogenic cell death [62,63,64,65,66]. Our data show upregulation of Bax and capsase-3 expression and downregulation of Bcl-2, indicating that apoptosis occurs via the extrinsic pathway as well [65]; a loss of JNK could primarily trigger extrinsic apoptosis. Moreover, Bax/Bcl-2/caspase-3 is also involved in other types of regulated cell death, including immunogenic cell death, mitotic catastrophe, and mitochondrial permeability transition (MPT)-driven necrosis [67], thus suggesting JNK inhibition to mediate Bax/Bcl-2/caspase-3 apoptosis. We herein showed loss of JNK, which is in concordance with a study by Wang et al., in breast cancer where overexpression of JNK did not cause apoptosis and correlated with poor prognosis [45]. Moreover, while activation of JNK results in loss of Bcl-2 expression [68,69,70], the mechanism is controversial as Bcl-2 phosphorylation enhances cell survival signaling [68,71,72,73,74], thus making the role of Bcl-2 phosphorylation in JNK-stimulated apoptosis nascent. Moreover, studies have demonstrated that JNK activation does not result in Bcl-2 phosphorylation [59,75], thus indicating that JNK might regulate another kinase or phosphatase resulting in Bcl-2 phosphorylation.

Although the roles of the Bcl-2 family of proteins in JNK-dependent apoptosis remain nascent, the results of the current study indicate that the proapoptotic Bax subfamily of Bcl-2-related proteins is not essential for JNK-dependent apoptosis. These data demonstrate a dual role of JNK in carcinogenesis which can be both oncogenic and tumor suppressive, as indicated previously. Alternatively, JNK activity can be tissue-specific and cell-type-dependent, differing based on tumor stage and status, as well as the presence of activated upstream and downstream molecules and stress signals [76,77,78,79,80]. Nevertheless, further work is needed to unravel the complexity of the interaction of JNK pathway and its molecules, to help pave the way for the development of anticancer therapeutic strategies. Moreover, in our laboratory, we are aiming to derive the active compounds of *EA* that can plausibly be involved in the inhibition of cancer progression.

## 4. Materials and Methods

### 4.1. Plant Collection and Extract Preparation

*EA* flowers were obtained during the second week of June, from Montreal, Quebec, Canada, and were dried and stored in a dark place, at room temperature, as previously described [12]. The extract was prepared by boiling 3 g of finely grounded dry *EA* flowers per 100 mL of autoclaved distilled water, at 150 °C, on a hot plate, for 20 min, with continuous stirring. The flower extract solution was then filtered, using a 0.45 μm filter unit, and stored at 4 °C until use. Dilutions were prepared in cell culture media for various applications. For each experiment, the extract was freshly prepared.

### 4.2. Cell Culture

Two different human HER2-positive breast cancer cell lines (SKBR3 and ZR75-1) derived from females were obtained from American Type Culture Collection (ATCC) (Rockville, MD, USA). Cell lines were grown and expanded in RPMI-1640 (Gibco, Life Technologies) supplemented with 10% fetal bovine serum (Gibco, Life Technologies, Massachusetts, MA, USA), 2 mM l-glutamine, 1% PenStrep antibiotic (Invitrogen, Life Technologies, Carlsbad, CA, USA) at 37 °C, and 5% CO_2_ humidified atmosphere. Human normal mammary epithelial cells immortalized by E6/E7 of HPV type 16 (HNME-E6/E7) were used to assess plant extract toxicity [81]. Cells were maintained in Gibco^®^ Keratinocyte-SFM (1X) media (Gibco, Life Technologies). All the experiments were carried out when cells were ~70–80% confluent.

### 4.3. Cell Viability Assay

HER-2-positive breast cancer cell lines, SKBR3 and ZR75-1, were seeded on clear bottom 96-well plates (10,000 cells/well) and cultured in RPMI-1640 supplemented with 10% fetal bovine serum (FBS) and 1% penicillin and streptomycin (100 µL/well).

*Elaeagnus angustifolia* (*EA*) solution was used to treat cells at different concentrations (25, 50, 75, 100, 150, and 200 µL/mL) for a period of 48 h. Control wells received 100 μL of media (control). The inhibition of cell viability was determined, using Alamar Blue Cell viability reagent (Invitrogen, Thermo Fisher Scientific, Waltham, MA, USA), according to the manufacturer’s protocol. The shift in fluorescence was measured at 570 nm (excitation) and 600 nm (emission), in a fluorescent plate reader (Infinite M200, Tecan, Grödig, Austria), after 4 h of incubation with the dye. Relative cell proliferation was determined based on the fluorescence of *EA*-treated cells relative to that of control cells.

### 4.4. Cell Cycle and Apoptosis Assay

SKBR3 and ZR75-1 cells (1 × 10^6^ cells/dish) were plated in 100 mm Petri dishes, with overnight incubation. The cells were then starved with serum-free RPMI-1640 medium for a period of 6–12 h to synchronize the cells into the G_0_ phase of the cell cycle. Synchronized cells were then treated with *EA* extract (100 and 200 µL/mL) for 48 h. Cells were harvested, washed twice with PBS, fixed overnight in 70% ice-cold ethanol, and, subsequently, their DNA was stained with 50 μg/mL FXCycle PI/RNase staining solution (Invitrogen, Thermo Fisher Scientific) after RNase A treatment (50 μg/mL) (Thermo Fisher Scientific), at 37 °C, for 30 min, according to standard protocol [12]. Cell-cycle analysis was performed by flow-cytometry (BD Accuri C6, BD Biosciences, USA), and cells in G_0_/G_1_, S, G_2_/M and the sub-G_0_/G_1_ (apoptotic) phases were quantified by using FlowJo software.

Furthermore, for apoptosis assay, the Annexin V-fluorescein isothiocyanate (FITC)/7-amino-actinomycin D (7-AAD) Apoptosis Kit-559763 (BD Biosciences, USA) was used as per the manufacturer’s instructions. Briefly, cells (1 × 10^6^ cells/dish) were seeded into 100 mm culture dishes and were maintained overnight in a medium containing 10% fetal bovine serum. The cells were collected by trypsinization and washed with phosphate buffered saline (PBS). Then, cells were resuspended in 200 µL of binding buffer. Annexin V staining was accomplished following product instructions (Clontech, Palo Alto, CA). In brief, 5 µL Annexin V-FITC and 5 µL 7-AAD were added to the samples for 15 min in the dark. However, for controls (unstained cells), they were stained with PE Annexin V (no 7-AAD) as well as with 7-AAD (no PE Annexin V). The cells were analyzed by flow cytometry (BD Accuri C6, BD Biosciences, San Jose, CA, USA). Data were presented as density plots of Annexin V-FITC and 7-AAD staining.

### 4.5. Cell Invasion Assay

Cell invasion assay was carried out in 24-well Biocoat Matrigel invasion chambers (pore size of 8 µm, Corning, USA) as per manufacturer’s protocol. In brief, the bottom chamber was filled with RPMI-1640 medium, and the upper chamber was seeded with untreated, as well as treated, cells (5 × 10^4^ cells), and then incubated at 37 °C. After 24 h incubation, non-invasive cells were scraped with a cotton swab, and cells that migrated to the lower surface of the membrane were fixed with methanol and stained with 0.4% crystal violet. For quantification, cells were counted under the Leica DMi1 inverted microscope (Leica Microsystems, Wetzlar, Germany) in five predetermined fields, as previously described [81]. Percentage inhibition of invasive cells was calculated with respect to untreated cells. Each experiment was carried out in triplicates.

### 4.6. Soft Agar Colony Formation Assay

Next, we determined the number of colonies formed prior and post-treatment, using soft agar growth assay. A total of 2 × 10^3^ cells of SKBR3 and ZR75-1 were placed in their medium containing 0.2% agar with/without 100 and 200 µL/mL of *EA* extract (treated and control cells, respectively) and plated in a 6-well plate covered with a layer of 0.4% agar prepared in RPMI-1640 medium. Colony formation was examined every 2 days for a period of 2 weeks. Colonies in each well were counted, using the Leica SP8 UV/Visible Laser confocal microscope (Leica Microsystems, Wetzlar, Germany).

### 4.7. Western Blot Analysis

We analyzed the expression levels of proteins involved in the molecular pathways, such as apoptosis by Western blot analysis, as previously described by our group [81]. Briefly, SKBR3 and ZR75-1 cells (1 × 10^6^ cells) were seeded and treated with *EA* extract (100 and 200 µL/mL) for 48 h. Cell lysates were collected, and equal amounts of protein (30 μg) were resolved on 10% polyacrylamide gels and electroblotted onto PVDF membranes. The PVDF membranes were probed with the following primary antibodies: anti-mouse E-cadherin (AbcamID#: ab1416), anti-rabbit β-catenin (CST 9562), anti-rabbit phosphorylated β-catenin (CST 4176), anti-rabbit Vimentin (Abcam: abID# 92547), anti-rabbit Fascin (AbcamID#: ab183891), anti-mouse Bax (ThermoFisher Scientific: MA5-14003), anti-mouse Bcl-2 (Abcam: abID# 692), anti-rabbit Caspase-3 (Abcam: abID# 13847), anti-mouse ErbB2 (Abcam: abID# 16901), anti-rabbit phosphorylated ErbB2 (Abcam: abID# 47262), anti-rabbit JNK1/JNK2/JNK3 (Abcam: abID# 179461), and anti-rabbit phosphorylated-c-Jun (Ser73) (Cell Signal Technologies, ID# 9164). To ensure equal loading of protein samples, the membranes were re-probed with anti-mouse β-actin (Abcam: abID# 6276).

Immunoreactivity was detected by using ECL Western blotting substrate (Pierce Biotechnology, Rockford, IL, USA), as described by the manufacturer.

In order to obtain a relative quantification of protein expressions, images acquired from Western blotting were analyzed, using ImageJ software. The intensity of the bands relative to the β-actin bands was used to calculate a relative expression of proteins in each cell line.

### 4.8. Statistical Analysis

The data were presented as mean ± SEM from three independent experiments performed in triplicates, and a t-test was used to compare the difference between treated and untreated cells. To evaluate significance for cell cycle, a Chi-square test was performed to compare significance between the different phases. Data were analyzed by using GraphPad Prism software (version 8.4.3), and differences with *p* < 0.05 were considered significant.

## 5. Conclusions

To the best of our knowledge, this is the first report, on the effect of *EA* in HER2-positive breast cancer and its underlying mechanism. Furthermore, this study brings about novel therapeutic potential by demonstrating the induced inhibition of HER2 and JNK activation by *EA* plant extract in human breast cancer cells. Our study points out that the downregulation of JNK can be one of the molecular pathways responsible of increasing E-cadherin and β-catenin and decreasing the expressions of vimentin and fascin. This is an interesting finding, since it can be potentially used as a target to inhibit cell invasion of HER2-positive breast cancer cells by reversing EMT or inducing RELT. In parallel, our data also demonstrate that high consecrations of *EA* trigger apoptosis, particularly in breast cancer cells, which is associated with Bcl-2/Bax/caspase-3 signaling pathway in HER2-positive cancer cells. We believe that *EA* might act as a candidate therapeutic agent based on its anticancer activity which can pave the way for potential more advanced therapeutic approaches in breast cancer management, especially HER2-positive cases.
